# Clinical applications of polygenic breast cancer risk: a critical review and perspectives of an emerging field

**DOI:** 10.1186/s13058-020-01260-3

**Published:** 2020-02-17

**Authors:** Tatiane Yanes, Mary-Anne Young, Bettina Meiser, Paul A. James

**Affiliations:** 1 Psychosocial Research Group, Prince of Wales Clinical School, Faculty of Medicine, UNSW Sydney, Sydney, NSW Australia; 2 The University of Queensland Diamantina Institute, Dermatology Research Centre, University of Queensland, Brisbane, QLD 4102 Australia; 3 Parkville Integrated Familial Cancer Centre, Peter MacCallum Cancer Centre, Melbourne, VIC 3000 Australia; 4 Kinghorn Centre for Clinical Genomics, Garvan Institute of Medical Research, Sydney, NSW 2010 Australia

**Keywords:** Breast cancer, Polygenic risk score, Risk prediction

## Abstract

Polygenic factors are estimated to account for an additional 18% of the familial relative risk of breast cancer, with those at the highest level of polygenic risk distribution having a least a twofold increased risk of the disease. Polygenic testing promises to revolutionize health services by providing personalized risk assessments to women at high-risk of breast cancer and within population breast screening programs. However, implementation of polygenic testing needs to be considered in light of its current limitations, such as limited risk prediction for women of non-European ancestry. This article aims to provide a comprehensive review of the evidence for polygenic breast cancer risk, including the discovery of variants associated with breast cancer at the genome-wide level of significance and the use of polygenic risk scores to estimate breast cancer risk. We also review the different applications of this technology including testing of women from high-risk breast cancer families with uninformative genetic testing results, as a moderator of monogenic risk, and for population screening programs. Finally, a potential framework for introducing testing for polygenic risk in familial cancer clinics and the potential challenges with implementing this technology in clinical practice are discussed.

## Introduction

Breast cancer is a common disorder with a strong hereditary contribution. Women with a first-degree relative with breast cancer have a twofold increased risk of developing the disease [1, 2]. Much of the hereditary component of breast cancer remains unexplained, with pathogenic variants in moderate- and high-risk genes, such as *BRCA1* and *BRCA2* (*BRCA1/2*), accounting for less than 25% of the familial risk for the disease [3]. Recent studies suggest breast cancer is a complex disease, with polygenic factors accounting for a further 18% of the familial risk [4]. Currently, there is considerable debate over the clinical utility of polygenic information to assess breast cancer risk. Supporters of this technology argue that polygenic testing has the potential to provide risk information for a significant number of women who would otherwise receive uninformative genetic testing results and further stratify risk for those with pathogenic variants in high- and moderate-risk genes [5–7]. Additionally, polygenic risk promises to revolutionize population screening programs by providing personalized risk assessments and risk management strategies [8, 9]. However, it has also been argued that there is not enough evidence to support its implementation in clinical practice and that the test does not provide sufficient risk stratification to warrant implementation over traditional risk assessment tools [10–12]. Furthermore, there is no consensus for the implementation of this technology, such as which variants to include in the polygenic risk calculations. Despite these concerns, testing for polygenic breast cancer risk is now being implemented in clinical practice with several commercial genetic testing laboratory now offering the test [13, 14].

This article aims to review the evidence for testing for polygenic breast cancer risk and the different applications of the test, including for high-risk women with uninformative genetic testing results, as a modifier of monogenetic risk genes, and to guide population screening programs. This article also explores the potential benefits and limitations of this technology, as well as the challenges associated with implementing polygenic testing in clinical practice.

### Genome-wide association studies

There have been over 100 different breast cancer genome-wide association studies (GWAS), which have collectively led to the identification of over 182 variants associated with breast cancer risk at the genome-wide level of significance (Additional file 1) [15]. Given the small effect size of these variants, there has been an ongoing need to increase the sample sizes of GWAS to detect true genetic associations [16]. Increasingly large GWAS have been made possible due the establishment of international research consortia, including the Breast Cancer Association Consortium (BCAC) and The Collaborative Oncological Gene-Environment Study (COGS), formed in 2005 and 2009 respectively [17, 18].

The COGS combined data from multiple international consortiums with the goal of improving understanding of the genetic susceptibility to breast, ovarian, and prostate cancer, and designed a custom array, the Illumina iSelect (iCOGS) [18]. The first major breast cancer outcome from the COGS program was a GWAS that genotyped 52,675 cases and 49,436 controls of European ancestry [19]. Twenty-three of the 27 previously reported breast cancer associated loci were replicated using iCOGS and an additional 41 new breast cancer loci were identified. Further analysis of the full dataset suggested that more than 1000 loci are involved in breast cancer susceptibility [19].

In a follow-up study, a meta-analysis of 11 previous GWAS together with the data from the 41 studies genotyped in iCOGS was conducted. Data for 62,533 breast cancer cases and 60,976 controls of European ancestry was analyzed, leading to the identification of an additional 15 breast cancer associated variants along with replication of 71 of the 79 previously reported variants [20]. Consistent with previous GWAS, nearly all of these variants were located in intronic or intergenic regions of the genome, suggesting they are regulatory [20].

In the largest breast cancer GWAS to date, Michailidou et al. [4] genotyped 61,282 cases and 45,494 controls of European ancestry using the specifically developed Illumina OncoArray and results were also included in a meta-analysis of a further 61,695 cases and 60,480 controls from iCOGS and 11 other studies. This comprehensive analysis led to the identification of the 61 new breast cancer variants, and 49 out of the 102 previously reported loci showed evidence of association with breast cancer risk in the OncoArray dataset. Together, the 182 known loci were estimated to account for 18% of the familial risk for breast cancer [4].

There have been increasing efforts to conduct GWAS in non-European populations, with most of the reported breast cancer variants discovered in European cohorts [21–25]. Michailidou et al. [4] conducted a meta-analysis of 14,068 cases and 13,104 controls of East Asian ancestry. Out of 94 loci discovered in European populations, 50 showed evidence of association in the East Asian cohort. Similarly, five loci previously reported in Asian women showed evidence in the European cohort. However, such findings have not been consistent across different ethnic groups. For example, approximately 100 variants previously validated in European or Asian women did not provide comparable risk stratification in women of African ancestry, with 30 to 40% of variants conferring risk in one population but appearing to be protective in another [26]. These findings highlight the need to conduct further studies to assess the association of breast cancer variants in non-European populations and to identify novel variants that confer breast cancer risk in those populations.

Most GWAS to date have been unselected for breast cancer subtype and as a result include a majority of the more common estrogen receptor (ER) positive subtypes. Consequently, most of the reported loci have been more strongly associated with ER-positive tumors. For example, out of the 61 new variants reported by Michailidou et al. [4], 19 showed a specific association with ER-positive breast cancer, and only two were associated with ER-negative disease. There have been growing efforts to identify variants associated with other breast cancer subtypes. Using data from over 21,000 ER-negative cases, Milne et al. [27] reported on 125 variants specifically associated with ER-negative disease. Combined, these variants were estimated to account for 16% of the familial risk for this disease subtype [27]. A strong association between ER-negative variants and breast cancer risk in *BRCA1* mutation carriers was also reported [27]. Similarly to other GWAS, ER-negative variants have been mostly identified in European populations.

### Polygenic risk scores

Individually, each breast cancer SNP has a minimal effect on breast cancer risk. However, their combined effect, in the form of polygenic risk scores (PRS), has been shown to provide a degree of risk discrimination that can be used to stratify individuals into different categories of disease risk. Studies examining the effect of PRS on breast cancer risk have consistently reported a higher PRS among women diagnosed with the disease when compared to population controls (Table 1) [7, 30–32, 39–42]. Overall, studies of European populations have reported at least a twofold difference in breast cancer risk between the lowest and highest quartile of PRS distribution. Similar findings have also been reported across other populations including women of African American and Asian ancestry (Table 1) [26, 30, 33, 35, 36, 43]. However, not all studies have weighted the breast cancer variants for their specific ethic group, and therefore findings may not be an accurate representation of breast cancer risk for their population [35]. An increased rate of contralateral breast cancer has also been reported for women with a PRS in the highest quartile of risk distribution [7, 37, 44].

**Table 1:** Summary of PRS and breast cancer risk studies

Reference	Year	Study design	Cohort	Cohort location	Sample size	No. of SNPs	AUC (95% CI)	Risk prediction
Hüsing et al. [28]	2012	Retrospective	Non-familial	USA, Europe	6009 cases 7827 controls	32	0.58 (0.57–0 .60)	–
Sawyer et al. [7]	2012	Retrospective	High-risk families	Australia	1143 cases 892 controls	22	0.65 (0.63–0.68)	4.5-fold increased risk between lowest to highest quartile Lifetime risk: 6 to 27% for lowest and top quartile
Mavaddat et al. [29]	2015	Retrospective	Non-familial	USA, Canada, Australia, Europe	33,673 cases 33,381 controls	77	0.62 (0.62–0.63)	Threefold increased risk between fist centile and middle quintile Lifetime risk: 3.5 to 29% for lowest centile to highest centile
Shieh et al. [30]	2016	Retrospective	Non-familial	USA Caucasian	387 cases 387 controls	83	0.59 (0.56–0.53)	Twofold increased risk between lowest and higher quartile
				Asian American	51 cases 51 controls	76	0.64 (0.53–0.74)	
Evans et al. [31]	2016	Retrospective	High-risk families	England	364 cases 1605 controls	18	0.59 (0.55–0.63)	Twofold increased risk between lowest to highest quintile
Muranen et al. [32]	2016	Retrospective	High-risk families	Finland	427 cases 1272 controls	75	Not reported	1.55 odds ratio per unit increase in the PRS within the family dataset
Wen et al. [33]	2016	Retrospective	Non-familial	East Asia	11,760 cases 11,612 controls	88	0.60 (not reported)	2.26-fold increased risk between lowest and top quartile
Li et al. [34]	2017	Prospective	High-risk families	USA, Australia, Canada	2869 unaffected 1496 affected	24	0.59 (0.55–0.63)	Threefold increased risk lowest and highest quintile Lifetime risk: 21 to 51% for lowest and highest quintile
Hsieh et al. [35]	2017	Retrospective	Non-familial	Taiwan	446 cases 514 controls	6	0.60 (not reported)	2.26-fold increased risk between lowest and highest quartile
Khera et al. [9]	2019	Retrospective	Non-familial	UK Biobank	6586 cases 157,895 controls	5218 (LDpred)	0.69 (0.68–0.69)	–
Chan et al. [36]	2018	Retrospective	Non-familial	Singapore-Chinese	301 cases 243 controls	51	0.57 (0.51–0.61)	1.88-fold increased risk between the lowest and higher quartile
Wang et al. [37]	2018	Retrospective	Non-familial	African	1657 cases 2029 controls	34	0.53 (0.51–0.55)	4.74-fold increased risk between lowest percentile and top percentile
Mavaddat et al. [38]	2019	Prospective	Non-familial	USA Caucasian	11,428 cases 18,323 controls	305	0.63 (0.63-0.65)	4.37-fold increased risk between middle quintile and highest 1% Lifetime risk of ER-positive disease: 2 to 31% for lowest centile to highest centile Lifetime risk of ER-negative disease: 0.55 to 4% for lowest centile to highest centile

*CI* confidence interval

#### Polygenic risk and breast cancer classification

Polygenic risk scores have been shown to differentiate risk for ER-positive and ER-negative disease. However, as noted, PRS is more effective at stratifying the risk of ER-positive disease [26, 28, 29, 32, 33, 38, 41, 45–52]. Recently, Mavaddat et al. [38] developed a PRS that was optimized for prediction of breast cancer-specific subtype. This was achieved by weighting a subset of variants according to subtype-specific effect sizes, with the remaining variants weighted for overall breast cancer risk. Improvements in risk prediction for breast cancer-specific subtypes were reported. However, prediction of ER-positive disease was still superior to ER-negative disease; odds ratio (OR) per 1 standard deviation (SD) of the PRS was 1.68 and 1.45, respectively [38].

The possibility that PRS may provide information on prognostic factors has also been examined. In one study, a high PRS was associated with an increased risk of being diagnosed with breast cancer during routine screening rather than between screening (interval breast cancer) [48]. When compared to breast cancer diagnosed during routine screening, interval cancers are associated with poorer prognosis and more aggressive tumors [48]. Similarly, two studies reported that a high PRS was associated with more favorable tumor characteristics including lower-grade ER-positive breast cancer, smaller size tumor, and less likely to be diagnosed with distant metastasis [45, 53]. However, these findings have not always been consistently replicated in other studies [54].

#### Accuracy of polygenic risk models

Several studies have assessed the discriminatory accuracy and calibration of breast cancer PRS. The discriminatory accuracy of PRS has been most commonly assessed by calculating the area under the receiver operating characteristic curve (AUC) (Table 1). The AUC is the overall probability that the predicted risk is higher for cases than for controls. Values range from 0.5 (risk for cases is higher 50% of the time, indicating the model does not discriminate cases from controls) to 1.0 (risk higher for cases 100% of the time, thus perfect discrimination) [55]. The reported AUC for breast cancer PRS has been modest, ranging from 0.58 to 0.65 for European populations and 0.53 to 0.64 for non-European populations (Table 1). Accuracy of PRS has also been reported based on breast cancer subtype, with studies reporting higher AUC for ER-positive cancer when compared to ER-negative disease [38, 46, 56].

Calibration (i.e., an assessment of how well the model’s predicted probability agrees with observed risk) was assessed in two studies, with both reporting excellent calibration for a PRS based on just 18 variants for a cohort of European ancestry [31, 41]. In another study, PRS calculated based on 75 variants was shown to be a well calibrated model for women of African American and Hispanic ancestry [39]. Finally, a study examining the effect of including PRS for risk assessment reported risk re-classification for 53% of their cohort compared to using the Manchester Scoring System alone [31], with 25% of women moving into a higher-risk category and 27% into a lower-risk category.

Determining the appropriate number of variants to include in the PRS calculation has remained a significant challenge. However, it is evident that increasing the number of variants has only had a minimal impact on the accuracy of PRS. For example, Mavaddat et al. [29] reported an AUC of 0.62 for a PRS based on 77 variants; in contrast, a PRS based on 313 variants had an AUC of 0.63 [38]. Despite the limited changes in AUC, there is evidence of improvements in the ability of the model to distribute risk in the population. Specifically, OR per 1 SD of the PRS was 1.46 for the 77 PRS [29], compared to a OR of 1.61 for the 313 PRS [38]. The modest improvement in AUC is to some extent expected with the progressive increase in the size and power of GWAS. This contributes to at least two moderating effects. Firstly, as the power of GWAS improves, variants are detected that confer lower breast cancer risk than in earlier smaller studies [57]. As a result, the additional variants that are incorporated in the PRS, add less to the overall prediction of breast cancer risk compared to the initially discovered variants in a “diminishing returns” effect. Secondly, the replication of individual SNPs or risk loci in larger GWAS allows for improved accuracy in the estimate of associated OR and can overcome the effect known as the “winners curse,” whereby the first report of a SNP association tends to provide an inflated estimate of the associated risk. As a result, early estimates of the discrimination of the PRS will have a tendency for overestimation, an effect that will be corrected over time as the OR for each SNP tends to be revised downward. These problems are addressed in more recent studies that have used new statistical methods of calculation polygenic risk with the goal of improving risk prediction, including creating a meta-PRS [42] and using LDpred [9]. Nevertheless, further research is still needed to determine the best-performing PRS.

#### Polygenic risk in combination with risk prediction models

Several studies have examined the effect of PRS on existing risk prediction models, including the Gail Model [39, 46, 56, 58–62], Breast and Ovarian Analysis of Disease Incidence and Carrier Estimation Algorithm (BOADICEA) [46, 63, 64], Tyrer-Cuzick (TC) [39, 41, 46, 49, 52], BRCAPRO [46], and Breast Cancer Surveillance Consortium (BCSC) [30, 40, 65] (Table 2). These statistical models utilize a range of well-known breast cancer risk factors such as personal and family history, breast pathology, and lifestyle factors to estimate the risk of breast cancer.

**Table 2:** Summary of studies assessing PRS and breast cancer risk prediction models

Reference	Year	Study design	Cohort	Cohort location	Sample size	No. of SNPs	Risk prediction model	AUC (95% CI) model only	AUC (95% CI) PRS only	AUC (95% CI) model and PRS
Wacholder et al. [58]	2010	Retrospective	Non-familial	USA, Poland	5590 cases 5998 controls	10	Gail	0.58	0.60	0.62
Mealiffe et al. [59]	2010	Retrospective	Non-familial	USA	1664 cases 1636 controls	7	Gail	0.54 (0.52–0.57)	0.59 (0.56–0.61)	0.62 (0.59–0.64)
Darabi et al. [60]	2012	Retrospective	Non-familial	Sweden	1569 cases 1730 controls	18	Gail, BMI, and PD	0.60 (0.58–0.62)	0.59 (0.56–0.61)	0.62 (0.59–0.64)
Dite et al. [56]	2013	Retrospective	High-risk families	Australia	962 cases 463 controls	7	Gail	0.58 (0.55–0.61)	0.58 (0.54–0.61)	0.61 (0.58–0.64)
Allman et al. [39]	2015	Retrospective	Non-familial	Hispanic	147 cases 3201 controls	75	Gail	0.55 (0.51–0.60)	0.59 (0.54–0.64)	0.61 (0.56–0.66)
							TC	0.53 (0.48–0.57)		0.59 (0.54 to 0.64)
				African American	421 cases 7049 controls	75	Gail	0.56 (0.53–0.59)	0.59 (0.54–0.64)	0.59 (0.56–0.61)
							TC	0.51 (0.48–0.54)		0.55 (0.52–0.58)
Vachon et al. [40]	2015	Retrospective	Non-familial	USA	1643 cases 2397 controls	76	BCSC/BI-RADS	0.66 (0.61–0.70)	0.68 (0.66–0.69)	0.69 (0.67–0.71)
Dite et al. [46]	2016	Retrospective	High-risk families	Australia	1223 cases 805 controls	77	BOADICEA	0.66 (0.63–0.70)	0.61 (0.58–0.65)	0.70 (0.67–0.73)
							BRCAPRO	0.65 (0.62–0.68)		0.69 (0.66–0.72)
							Gail	0.64 (0.60–0.68)		0.67 (0.63–0.70)
							TC	0.57 (0.53–0.60)		0.63 (0.59–0.66)
Shieh et al. [30]	2016	Retrospective	Non-familial	Combined cohort	482 cases 483 controls	83	BCSC	0.62 (0.59–0.66)	0.60 (0.57–0.64)	0.65 (0.61–0.68)
				USA Caucasian	387 cases 387 controls	83	BCSC	0.62 (0.59–0.66)	0.59 (0.56–0.63)	0.63 (0.59–0.62)
				Asian American	51 cases 51 controls	76	BCSC	0.62 (0.59–0.66)	0.64 (0.53–0.74)	0.72 (0.62–0.82)
Shieh et al. [65]	2017	Retrospective	Non-familial	USA	110 cases 214 controls	86	BCSC and estradiol levels	0.67 (0.60–0.74)	0.68 (0.61–0.75)	0.72 (0.65–0.79)
Starlard-Davenport et al. [62]	2018	Retrospective	Non-familial	African American	319 cases 599 controls	75	Gail	0.65 (0.61–0.69)	0.58 (0.54–0.62)	0.65 (0.62–0.70)
Van Veen et al. [41]	2018	Prospective	Non-familial	England	8897 unaffected 466 affected	18	TC and breast density	0.58 (0.52–0.62)	Not reported	0.64 (0.62–0.71)
Zhang et al. [61]	2018	Prospective	Non-familial	USA	4006 cases 7874 controls	67	Gail, breast density, hormone levels	0.56* (0.55–0.57)	Not reported	0.65 (0.64–0.66)
Evans et al. [52]	2019	Prospective	Non-familial	England	9362 unaffected women	18	TC and breast density	Not reported	Not reported	Not reported
Lakeman et al. [64]	2019	Retrospective	High-risk families	Netherlands, Hungary	323 cases 262 unaffected female relatives	77	BOADICEA	Not reported	Not reported	Not reported
Läll et al. [42]	2019	Retrospective	Non-familial	Estonia-Biobank and UK Biobank	3474 cases 43,827 controls	Meta GRS	Gail model	0.68	0.64	0.72

*CI* confidence interval, *BMI* body mass index, *PD* percentage density

*AUC for Gail model only, not inclusive of breast density and hormone levels

Currently, all studies of European populations have reported improved accuracy when PRS is added to the model (Table 2). In a study of four different risk prediction models, Dite et al. [46] reported that across the different approaches, approximately 10% of women with a personal history of breast cancer moved into a higher breast cancer risk category and less than 2% into a lower-risk category (Table 2). Similarly, Li et al. [34] found that adding PRS to BOADICEA resulted in 16% of their cohort moving above the risk threshold for breast screening through magnetic resonance imaging (MRI). Currently, the highest AUC has been reported in a model that combined BCSC, circulating estradiol levels, and PRS (AUC = 0.72) [65]. The BCSC model uses information on age, ethnicity, first-degree family history of breast cancer, and breast density to assess breast cancer risk [65]. Such findings highlight the potential for improving current breast cancer risk discrimination through more comprehensive prediction models that include genomic information, although even the best models described to date leave room for continued improvement. As in other areas, there is a lack of studies examining these approaches in women of non-European ancestry; three relatively small studies have examined the combination of the Gail model and PRS in African American, Hispanic and Asian women respectively [30, 39, 62] providing some evidence of improved risk prediction in these groups.

A limitation of many of these studies is that they assume independence between the PRS and previously described risk factors. Ignoring the correlation between components in the model, most obviously between genetically determined risk and family history, is likely to result in an overestimation of risk among women with a family history of the disease. A recent study by Lee et al. [66] has addressed this issue in the BOADICEA model where the effect of the residual family history is attenuated by the PRS. Future studies should aim to incorporate similar features into the other risk prediction models to address to the redundancy between family history and PRS.

### Applications of polygenic breast cancer risk

Despite earlier concerns, the latest research has demonstrated that PRS is a strong predictor of breast cancer risk, with most studies of women from European ancestry suggesting a greater than twofold difference in risk between the lowest and highest PRS quartiles (Table 1). Consideration now turns to the clinical utility of PRS and challenges with clinical implementation of testing. There are several applications to polygenic breast cancer risk that include (i) to provide addition risk information to families at high-risk of breast cancer with uninformative genetic testing results, (ii) as a moderator of monogenic risk, and (iii) to guide population breast screening programs. Each application of polygenic testing has its own benefits and limitations which warrant further exploration.

#### Familial cancer clinic

Currently, most women at high-risk of breast cancer receive uninformative results from genetic testing of high- and moderate-risk genes. Uptake of breast cancer risk management strategies among this group of women is reported to be low [67, 68]. Thus, there is a need to develop new method of risk stratification to inform risk management decisions for women with uninformative results from monogenic testing. Studies assessing the application of polygenic factors for familial cancer have reported that PRS was predictive of breast cancer risk among women from high-risk families with uninformative *BRCA1/2* result [7, 31, 32, 34, 46, 56, 64]. Higher PRS has also been reported among women from breast cancer families when compared to those without a family history of the disease [29, 31, 32], suggesting that breast cancer variants may cluster in affected families. Despite some evidence of variant clustering, it is not possible to predict an individual’s PRS based on their relative’s result [69]. Additionally, it is likely that polygenic testing will be implemented as part of a risk prediction model such as Tyrer-Cuzick, and therefore, breast cancer risk should be evaluated individually regardless of their relatives’ risk level.

Polygenic testing is likely to be implemented in familial cancer clinics, alongside an assessment of family history, for women at increased risk of breast cancer with uninformative genetic testing results. Women with uninformative result and an increased PRS could be counseled about the higher risk of early-onset breast cancer, increased risk of contralateral breast cancer, no change in ovarian cancer risk, and reduced chance of a pathogenic variant in high-penetrance genes [7]. This information can be used to guide risk management decisions such as increased surveillance and chemoprevention. Currently, PRS is a better predictor of ER-positive breast cancer. Given that current risk-reducing medications, such as tamoxifen, function by interfering with the estrogen pathway, it is possible that this approach will be a particularly effective for women with a high PRS based on current variants [29]. Among women with a low PRS, the residual familial risk will be estimated through the integration of PRS into risk prediction models [66]. This group of women could also be informed about the ongoing risk for ER-negative disease.

Currently, there is no framework to support the implementation of breast cancer polygenic testing across clinical genetic services. Historically, genetic services have focused on testing of monogenic risk genes (e.g., *BRCA1/*2) and its familial implications. However, a shift towards a personalized model of care will be required as polygenic testing continues to be implemented into clinical practice. While genetic counseling for common complex disorders are available [70, 71], a new model of genetic counseling that accounts for both monogenic and polygenic risk, as well as additional risk factors such as family history and lifestyle factors, is yet to be developed. Additional training of genetic health professional will also be required to ensure clinicians are able to effectively communicate this information. Furthermore, there are few studies assessing communication and response to receiving PRS [72, 73]. Research is needed to assess how women understand this complex information and the psychological and behavioral impact of receiving polygenic risk. These challenges will be magnified as polygenic testing moves into mainstream medicine. Despite the challenges, testing for breast cancer PRS is now clinically available [13, 14], with testing being target to unaffected women at increased risk of familial breast cancer with uninformative results from monogenic testing.

#### Polygenic risk and single gene modification

Polygenic risk scores have been shown to modify risk associated with high- and moderate-risk breast cancer risk genes [6, 74]. Using a cohort of women from the Consortium of Investigators of Modifiers of *BRCA1/2* (CIMBA), Kuchenbaecker et al. [6] reported large differences in the absolute risk of developing breast cancer when PRS was incorporated in to the risk prediction model. Specifically, *BRCA1* carries with a PRS in the 10th percentile of risk distribution had a 56% chance of developing breast cancer by age 80 years. In comparison, those with a PRS in the 90th percentile had a 75% breast cancer risk by age 80 years. Evidence of subtype-specific PRS was also reported, with a PRS weighted for ER-negative risk displaying the strongest association with breast cancer risk in *BRCA1* carriers [6]. The AUC for these models were modest, with ER-negative PRS for *BRCA1* carriers having the highest discrimination (AUC = 0.58). In another study, a PRS based on 77 variants was a significant risk factors for CHEK2*1100delC carriers [74]. Early research on the impact of PRS on monogenic risk is promising [6, 74]. However, this literature is still limited to a few studies and therefore additional research is needed.

Additional research is also needed to determine the clinical outcomes associated with implementing PRS in familial cancer clinics and guidelines for risk management strategies will need to be developed. For example, risk-reducing strategies may be recommended to CHEK2*1100delC carriers with increased risk PRS, while increased surveillance may be an appropriate strategy for those with the same pathogenic variant and reduced risk PRS. Nevertheless, if early data is replicated, incorporating PRS to high- and moderate-risk gene testing would allow for personalization of risk prediction and ultimately facilitate risk management decisions for women with pathogenic variants in these genes.

#### Polygenic risk and population breast screening

Despite the strong evidence for the high variability of breast cancer risk within populations, most population screening programs only utilize age as a risk factor in determining recommendations for mammographic screening. However, there is growing evidence to support the inclusion of polygenic information to population screening programs, with studies reporting an age-specific effect for PRS [9, 29, 52, 63]. For example, Mavaddat et al. [29] reported women in the 99th percentile of PRS distribution reached the threshold for population screening in their early 30s, while women below the 20th percentile remained under the risk threshold for population screening up until age 70 years [29]. In another study, Khera et al. [9] estimated that 20% of the population would have a greater than twofold risk of developing breast cancer based on PRS alone. More recently, it was reported that greater levels of breast cancer risk stratification for the general population could be achieved by incorporating PRS, mammographic density, and other risk factors into the BOADICEA risk model [63]. The authors estimated that 13% of women in the population would be classified as moderate or high risk of developing breast cancer. Together, these data indicate that polygenic information has the potential to provide personalized risk management for a meaningful proportion of the population, including earlier and more frequent screening for women at increased risk and reduced mammographic screening for women with a lower PRS. Further research is now needed to evaluate the clinical utility of this personalized approach to population screening. For example, studies are needed to assess the extent in which PRS improves clinical outcomes, including reducing morbidity and mortality for at-risk women, and reducing overdiagnosis [75]. Studies are also needed to assess individual screening pathways including ages for commencing screening and modalities for calculating the absolute benefits of preventative strategies (e.g., chemoprevention) to allow women to make more informed choices about how to manage their level of risk.

Implementation of PRS into population screening programs also requires consideration of the social, ethical, and psychological outcomes. For example, consideration needs to be given to the acceptability of risk-based surveillance (particularly for those with a reduced risk), training of non-genetics health professionals, how best to communicate this information, and cost-benefit analyses [29, 75, 76]. Several large-scale studies are now underway to assess the impact of implementing PRS to breast screening program including the PROCAS [52, 77], CORDIS [78], and WISDOM trial [79]. These studies will provide a platform for the rigorous evaluation of risk-based screening, as well as a framework for the implementation of PRS into population screening programs. Nevertheless, PRS testing has the potential to change population screening programs and allow access to personalized genetic testing to a wider group of women, including testing of women with breast cancer unselected for family history, and women in the general population to tailor their mammographic screening schedule.

## Conclusion

Significant progress has been made in understating the genetic architecture of breast cancer and the role of polygenic information in familial breast cancer and for population screening programs. The studies presented in this review demonstrate that polygenic information has the power to effectively stratify breast cancer risk. These findings need to be considered in light of current limitations, including limited research on women from non-European ancestry and emphasis of ER-positive breast cancer. Equally, further research is needed to determine the best-performing PRS, with varying number of SNPs and statistical methods being currently used to calculate polygenic risk [9, 42]. Despite the current limitations, testing for polygenic risk is already being implemented in specialist familial cancer clinics to provide additional information for at-risk women with uninformative genetic test results, while research is ongoing to assess the clinical utility of PRS for population screening programs.

## Supplementary information

Additional file 1. Breast Cancer GWAS catalog summary dated 20.10.19. This file contains a summary of all the breast cancer GWAS registered in the GWAS Catalog as of the 20th October 2019.

## Data Availability

Data sharing is not applicable to this article as no datasets were generated or analyzed during the current study.

## Abbreviations

AUC: Area under the curve
BCAC: Cancer Association Consortium
BCSC: Breast Cancer Surveillance Consortium
BOADICEA: Breast and Ovarian Analysis of Disease Incidence and Carrier Estimation Algorithm
COGS: The Collaborative Oncological Gene-Environment Study
ER: Estrogen receptor
PRS: Polygenic risk scores
TC: Tyrer-Cuzick
